# Comparison of trend in chronic kidney disease burden between China, Japan, the United Kingdom, and the United States

**DOI:** 10.3389/fpubh.2022.999848

**Published:** 2022-09-06

**Authors:** Haoyu Wen, Donghui Yang, Cong Xie, Fang Shi, Yan Liu, Jiaming Zhang, Chuanhua Yu

**Affiliations:** ^1^Department of Epidemiology and Biostatistics, School of Public Health, Wuhan University, Wuhan, China; ^2^Department of Preventive Medicine, School of Health Sciences, Wuhan University, Wuhan, China; ^3^Institute of Preventive Medicine Information, Hubei Provincial Center for Disease Control and Prevention, Wuhan, China; ^4^Department of Pathology, The First Affiliated Hospital of Nanchang University, Nanchang, China; ^5^China Global Health Institute, Wuhan University, Wuhan, China

**Keywords:** chronic kidney disease, Burden of Disease, trend, decompose, incidence, death

## Abstract

Chronic kidney disease (CKD) caused heavy burden globally. This study aimed to investigate the patterns and temporal variations in the burden of CKD in China, Japan, the United Kingdom (U.K.), and the United States (U.S.) from 1990 to 2019, and decompose the difference in CKD disease burden between 1990 and 2019 into demographic factors. From 1990 to 2019, although the age-standardized rate (ASR) of incidence remained stable in the four countries, and the ASR of mortality and disability-adjusted life years (DALY) have declined in four countries (except for the increase in U.S.), the number of CKD incidence, death, and DALY increased significantly. The average disease burden per case in U.S. has increased between 1990 and 2019, with an increasing proportion of death-related disease burden. For the CKD due to diabetes and hypertension, whose incidences accounted for < 25% of the total CKD, while it accounts for more than 70% of the deaths (except in U.K. with 54.14% in women and 51.75% in men). CKD due to diabetes and hypertension should be the focus of CKD prevention and control. Considering the high treatment costs of CKD and ESRD, it is urgent and necessary to transform CKD treatment into primary and secondary prevention.

## Introduction

Chronic kidney disease (CKD) is defined by persistent urine abnormalities, structural abnormalities or impaired excretory renal function (1), which has caused heavy burden worldwide. In 2019, the number of patients with CKD in the world reached about 700 million, which is more than that of neoplasms, ischemic heart disease, stroke and chronic obstructive pulmonary disease (2). Approximately, 1.43 million people died from CKD in 2019, and this number is expected to rise by 2040 to 2.2 million in the best case scenario and 4 million in the worst case scenario (3). Moreover, CKD is predicted to rank as 5th in the leading causes of early death in 2040 (3). Recently, many studies have shown that CKD is associated with many diseases, such as hypertension, diabetes, and glomerulonephritis (4, 5). Great burden of hypertension and diabetes were observed that 1.13 billion (6) and 463 million people (7) suffered from hypertension and diabetes, respectively. Additionally, more glomerulonephritis may occur than in the past (8). Increases in cases of those disease drove growth in burden of CKD, which put enormous pressure on health-care resources. Additionally, CKD is also an important contributor to the poor outcome of non-communicable disease (9). Accordingly, it is critical to address the burden of CKD in order to achieve the United Nation's (UN) Sustainable Development Goal (SDG) that reduces premature mortality from non-communicable diseases (such as cardiovascular disease, chronic respiratory disease, diabetes, and cancer) by a third by 2030 (10).

The Global Burden of Disease (GBD) 2019 divides chronic kidney disease into 5 types: CKD due to diabetes mellitus type 1, CKD due to diabetes mellitus type 2, CKD due to glomerulonephritis, CKD due to hypertension, CKD due to other and unspecified causes. Many studies demonstrated that diabetes and hypertension were more common risk factors in CKD in developed countries (4), whereas glomerulonephritis and unknown causes accounted for more CKD in developing countries (5). The degree of development of a country is considered to be a strong social determinant of the disease burden of CKD (11). Therefore, studies on differences in the disease burden of CKD in countries with different levels of development are needed. China is a representative of developing countries, while the United Kingdom (U.K.) and the United States (U.S.) are representative developed countries, and Japan is a developed country with a similar culture and geographical location to China. Recently, many studies have estimated the burden of CKD nationally in China, Japan, the U.K., and the U.S. from economy and prevalence. In China, total cost spending on all patients with CKD in 2016 was 27,646 million RMB, constituting 6.50% of the overall expenditure, whereas the prevalence of CKD was only 4.86% among inpatients (12). In the U.S., the proportion of patients with CKD increased to 14.5%, and the expenditure spending on patients with CKD was over $120 billion in 2017 (13). Meanwhile, the prevalence of CKD has recently been 6.86% in the U.K. (14), while the prevalence of CKD has been 12.9% in Japan (15).

However, comprehensive comparative studies on the disease burden of CKD among the four countries remained sparse, and none of the existing studies on CKD explored the effect of demographic factors on changes in CKD disease burden, knowing this information could be useful for taking priority actions to address the CKD burden. Accordingly, in this study, we aimed to estimate the burden of CKD and examine its variations between 1990 and 2019 in China, Japan, U.K., and U.S. by using Global Burden of Disease Study 2019, and examine how sociodemographic progress has shaped the burden of CKD. We followed the Guidelines for Accurate and Transparent Health Estimates Reporting (GATHER; Supplementary material) (16).

## Materials and methods

### Data source

Data for this study were obtained from the latest GBD 2019 study (http://ghdx.healthdata.org/), which integrated heterogeneous data and estimate the burden for 369 diseases and injuries, 87 risk factors in 204 countries and territories by year, location, age, and sex from 1990 to 2019 (17, 18). GBD 2019 evaluated a health outcome according to the 9th and 10th revisions of the International Classification of Disease (ICD) and World Health Organization (WHO) clinical criteria and provided a comprehensive estimation [e.g., incidence, death, disability-adjusted life years (DALY)].

In this study, we obtained data on number and the age-standardized rate of the incidence, mortality, and DALY due to CKD from 1990 to 2019 in China, Japan, the U.K., and the U.S., which were processed by GBD through using cause of death ensemble modeling (CODEm) and DisMod-MR 2.1. All data used in the study were deidentified. Extensively detailed processes on estimation by GBD 2019 have been reported elsewhere previously (17, 18). DALY represents the overall burden due to years of healthy life lost, which consisted of two parts—years of life lost (YLL) and years of life disability (YLD). YLL resulted from premature death, while YLD was the health loss from living with non-fatal CKD sequelae. YLL was estimated by multiplying the number of death and remaining life expectancy at the age of death. Total non-fatal-disease-caused YLD was the sum of YLD of each sequela, which was calculated by multiplying the prevalence of each CKD sequela and its corresponding disability weight. The age-standard rate of incidence, death, and DALY were calculated by using the GBD 2019 global age-standard population.

We assessed all the uncertainty in all steps of estimating the process, including uncertainty in data sources and manipulations, measurement error, and the choice of the model. Number and the age- standardized rate of incidence, mortality, and DALY were reported with its 95% uncertainty interval (95% UI), which was calculated by taking 1,000 draws from the posterior distribution and bases on the ordinal 2.5th and 97.5th draws of uncertainty distribution.

### Methods

Since Das Gupta's method was published in 1993 (19), which has been used in numerous disease burden studies (20, 21). Changes in CKD DALY were decomposed into four drivers: population growth, changes in population age structures, changes in the prevalence rate, changes in case fatality and disease severity.

First, we express the CKD DALYs as the product of four factors:


$$\begin{array}{l} DALY_{y}=\;\sum_{a}pop\;size_{y}\;\cdot \frac{pop\;age_{a,y}}{pop\;size_{y}}\cdot \frac{prevalence_{a,y}}{pop\;age_{a,y}}\\ \;\cdot \frac{DALY_{a,y}}{prevalence_{a,y}} \end{array}$$

where, *a* is the age group and *y* is year.

For simplicity, let **Y** be the CKD DALYs by age; **S** be the size of the population (the first term on the right-hand side); **A** be the age structure of the population (second term); **P** be the age-specific prevalence rate for each age group (third term); **D** be the average DALYs of case for each age group (fourth term).

Then, we can simplify the identity for the two time periods of interest into:


$$\begin{array}{l} Y_{1990}=S_{1990}A_{1990}P_{1990}D_{1990}\\ Y_{2019}=S_{2019}A_{2019}P_{2019}D_{2019} \end{array}$$

To estimate the additive contribution of **S**, Das Gupta's method assumes that **A**, **P**, and **D** remain the same over time by applying a standardized rate of **A**, **P**, and **D**. This decomposition approach does not capture the interactions between factors. The additive contribution of population size, **S**, on the change in CKD DALYs between 1990 and 2019 is then calculated as:


$$\begin{array}{l} \;\left(S_{2019}-S_{1990}\right)\times \\ \left[\begin{array}{c} \frac{A_{2019}P_{2019}D_{2019}+A_{1990}P_{1990}D_{1990}}{4}+\\ \frac{\left(\begin{array}{c} A_{1990}P_{1990}D_{2019}+A_{1990}P_{2019}D_{1990}+A_{2019}P_{1990}D_{1990}+\\ A_{2019}P_{2019}D_{1990}+A_{2019}P_{1990}D_{2019}+A_{1990}P_{2019}D_{2019} \end{array}\right)}{12} \end{array}\right] \end{array}$$

where the latter term in square brackets are the **APD**-standardized rates for the two time periods. We follow the same step to estimate the contributions of **A**, **P**, and **D**.

## Results

### The trends of CKD incidence

**Figure 1:**
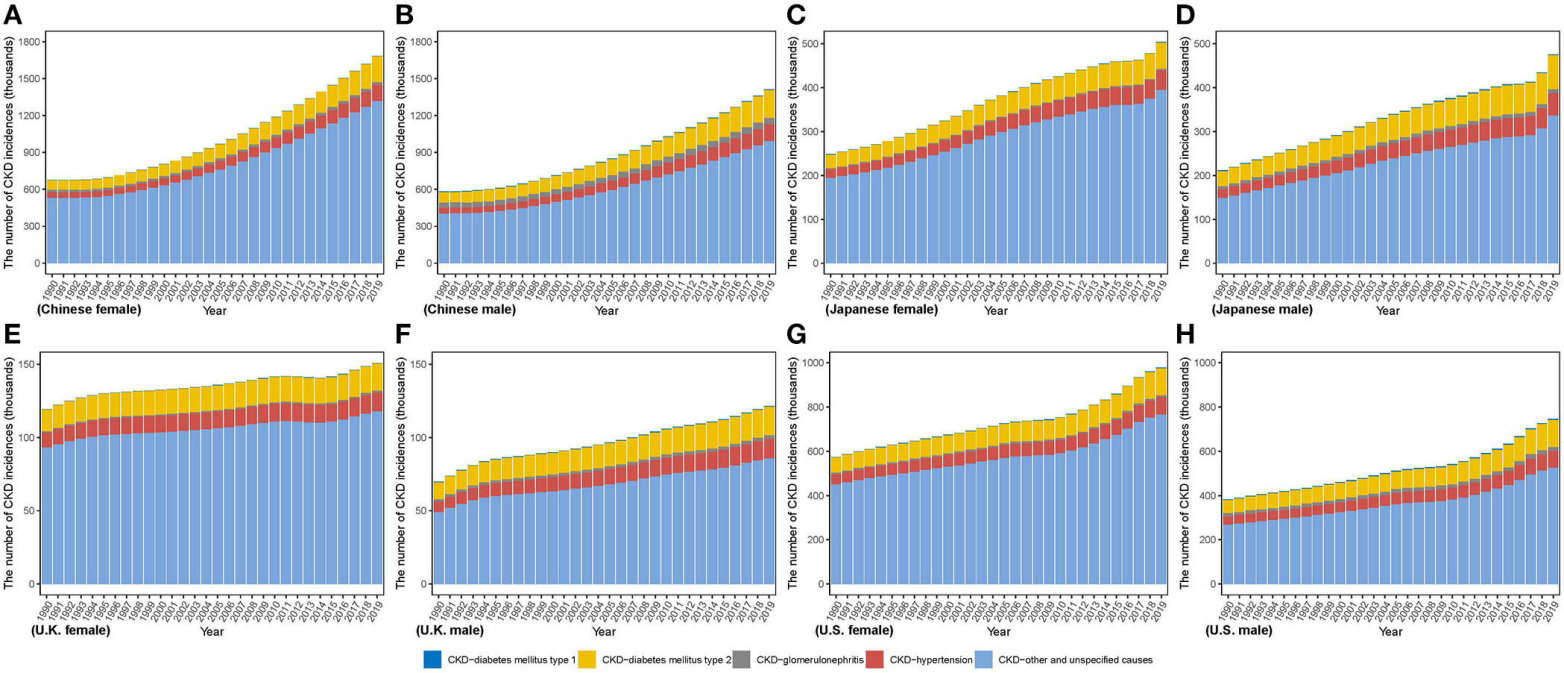
The number of CKD incidence cases from 1990 to 2019 in **(A)** Chinese women **(B)** Chinese men **(C)** Japanese women **(D)** Japanese men **(E)** U.K. women **(F)** U.K. men **(G)** U.S. women **(H)** U.S. men.

Figure 1 describes the steadily increasing trend of the CKD incidence case in both sexes from 1990 to 2019 in four countries. Overall, from 1990 to 2019, there were various increases in the incidence case in those four countries, relative increases ranging from 74.18% in U.K to 142.45% in China among men and from 26.37% in U.K. to 148.44% in China among women. Between 1990 and 2019, compared with men, women constituted more the incidence case in those all four countries. Through steady increase, in 2019, the highest incidence numbers in men and women were both in China [men: 1,413,861.63 (1,271,062.77, 1,565,254.87); women: 1,684,856.53 (1,539,008.17, 1,846,339.99)] while the lowest both in U.K. [men: 121,426.61 (108,972.87, 134,729.61); women: 150,749.89 (135,235.53, 166,389.66)]. In the four countries, the highest proportion of CKD types is CKD due to other and unspecified causes. CKD due to hypertension and CKD due to diabetes mellitus type 2 take a higher proportion of CKD incidence in men than in women.

**Figure 2:**
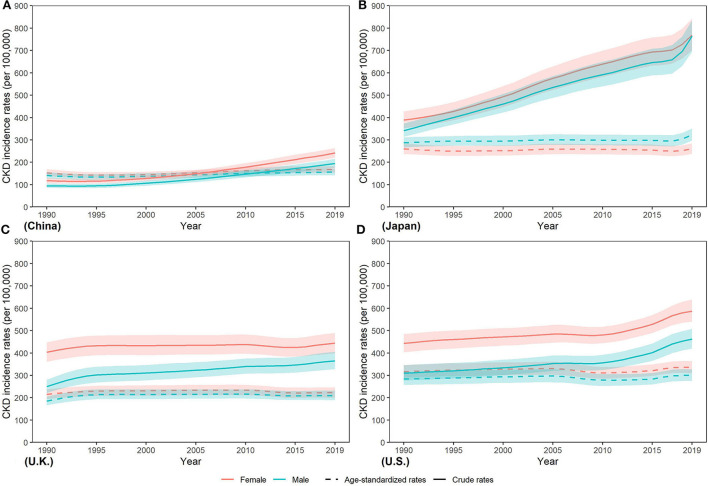
Trends in CKD incidence rates from 1990 to 2019 in **(A)** China, **(B)** Japan, **(C)** U.K., and **(D)** the U.S.

In contrast to the steep increase in incidence number, as Figure 2 and Supplementary Table 1 show, the age-standardized incidence rate (ASIR) only increased slightly in four countries from 1990 to 2019 in men and women, with the greatest change by 12.69% in U.K. and 10.00% in China, respectively, while the lowest change from 5.77% in U.S. and < 0.01% in Japan, respectively. In 2019, ASIR of CKD was higher in women than men in four countries except for Japan [men: 322.81 (296.77, 351.25) per 100,000 population; women: 260.17 (238.05, 284.35) per 100,000 population]. Moreover, ASIR in men and women was both lowest in China [men: 156.51 (142.15, 172.39) per 100,000 population; women: 168.28 (154.63, 183.25) per 100,000 population] while the highest ASIR in men and women was in Japan [322.81 (296.77, 351.25) per 100,000 population] and U.S. [335.44 (309.59, 364.23) per 100,000 population], respectively, which were about two times that of China. In 2019, China held the highest incidence number and the lowest incidence rates for CKD among the four countries.

### The trends of CKD death

**Figure 3:**
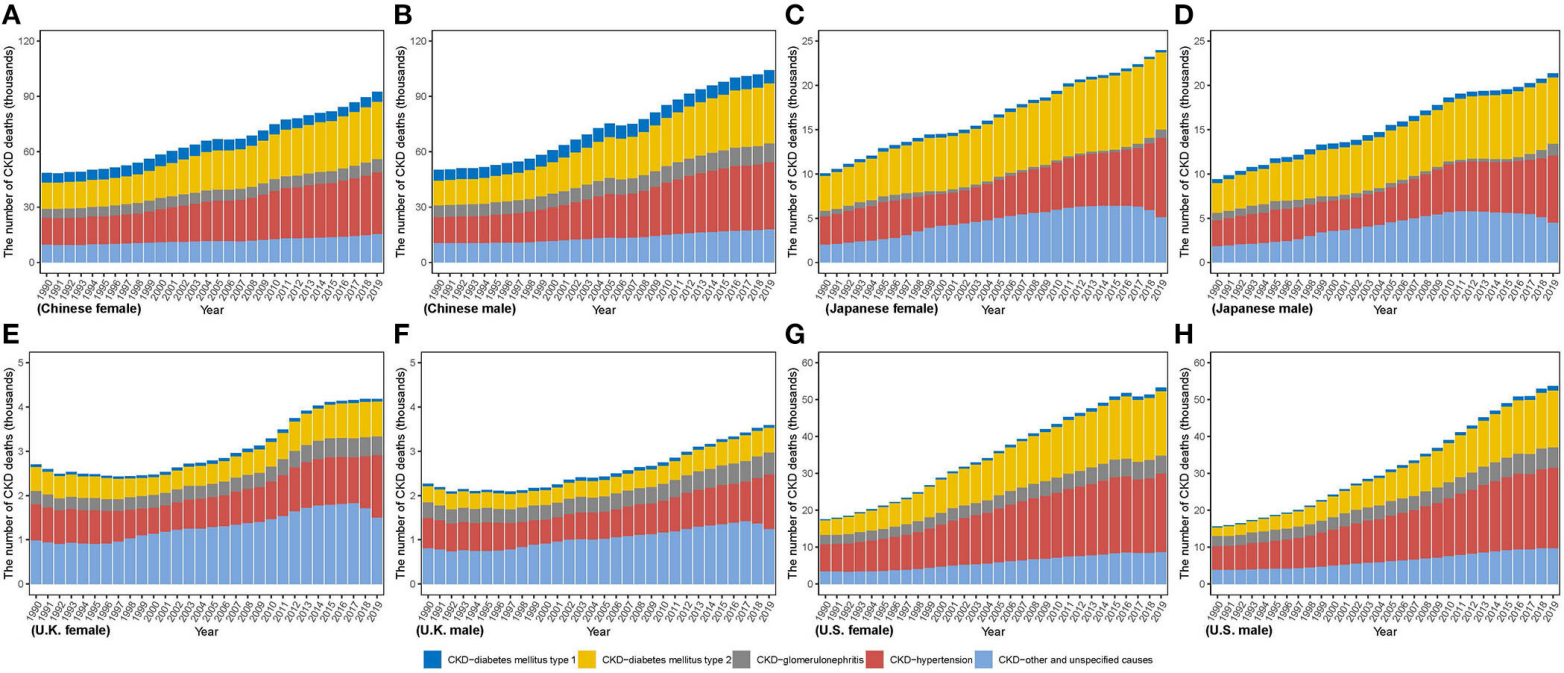
The number of CKD death cases from 1990 to 2019 in **(A)** Chinese women **(B)** Chinese men **(C)** Japanese women **(D)** Japanese men **(E)** U.K. women **(F)** U.K. men **(G)** U.S. women **(H)** U.S. men.

From 1990 to 2019, death number increased in all countries as same as incidence number (Figure 3). The lowest variation in both sexes was in U.K. (men: 58.53%; women: 54.73%), while the highest in U.S. (men: 244.23%; women: 204.29%). Between 1990 and 2019, more deaths in men were observed in China, whereas more deaths in women in Japan and U.K. In U.S., although men were responsible for more number in 1990, it appeared a reversal that number in women gradually exceeded that in men. In 2019, men comprised more death number in China and U.S., while more death in women occurred in Japan and U.K. Additionally, highest death numbers in men and women were both in China [men: 104,218.73 (84,554.90, 126,993.46); women: 92,507.51 (74,759.20, 111,027.53)], which were over twenty times greater than that in men [3,591.96 (3,259.10, 3,786.56)] and in women [4,183.60 (3,566.79, 4,553.87)] in U.K. CKD due to diabetes and hypertension accounts for a small proportion of the total CKD incidence but causes a large proportion of CKD deaths. For the CKD due to diabetes and hypertension, whose incidences accounted for < 25% of the total CKD, it accounts for more than 70% of the deaths (except in U.K. with 54.14% in women, and 51.75% in men). In 2019, the country with the highest proportion of CKD deaths due to hypertension in the total CKD deaths was U.S. (40.14% in women, 40.88% in men), and that for CKD due to diabetes mellitus type 1 and type 2 was China (5.91% in women, 6.92% in men) and Japan (34.90% in men, 36.23% in women), respectively.

**Figure 4:**
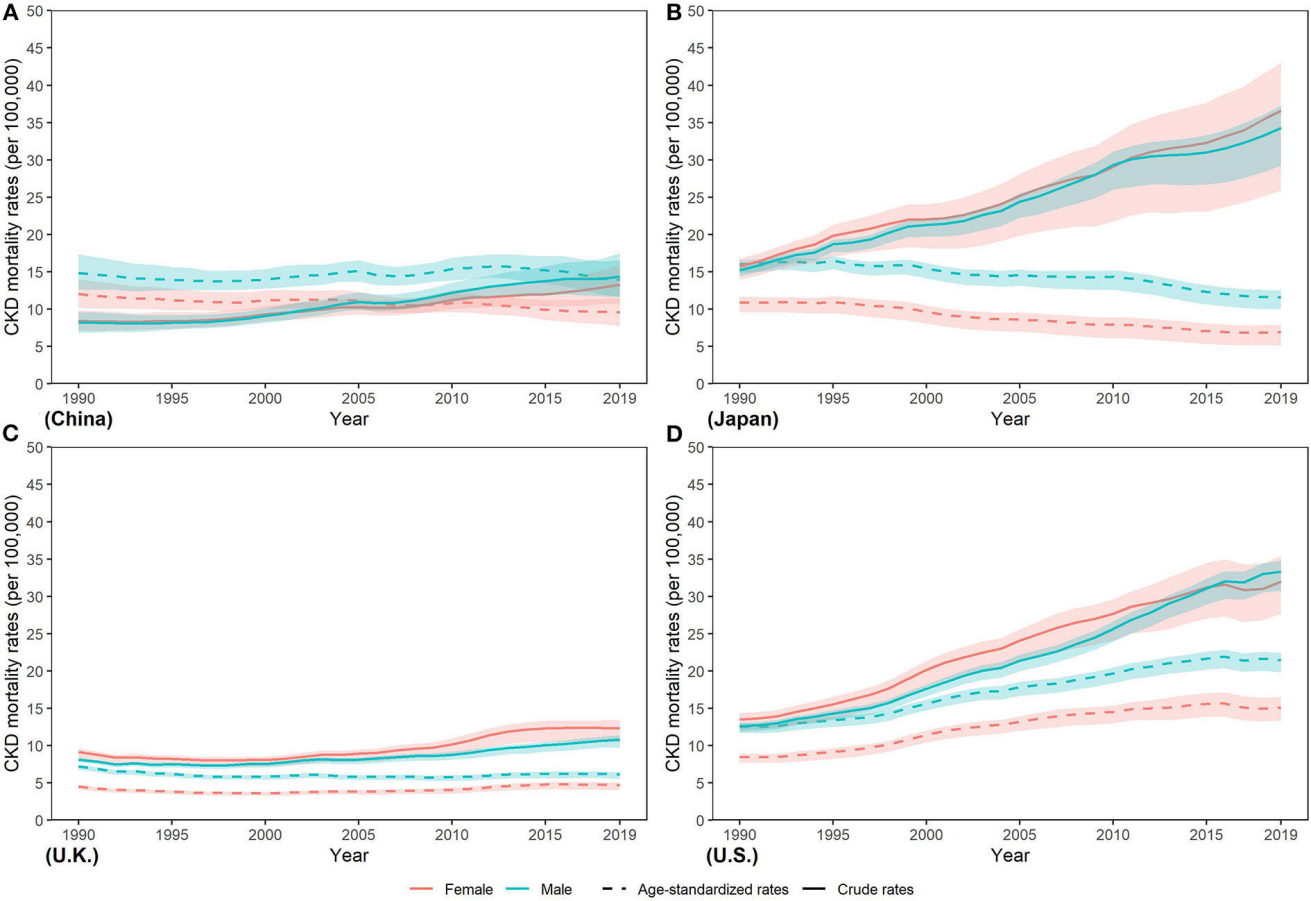
Trends in CKD mortality rates from 1990 to 2019 in **(A)** China **(B)** Japan **(C)** U.K., and **(D)** the U.S.

Furthermore, compared with the age-standardized mortality rate (ASMR) in women, ASMR in men was higher between 1990 and 2019 in all four countries (Figure 4). From 1990 to 2019, ASMR decreased by various percents in both sexes in China (men: −6.06%; women: −20.57%) and Japan (men: −27.58%; women: −36.89%) and men in U.K. (−15.16%), while ASMR increased steadily in both sexes in U.S. (men: 70.92%; women: 78.70%) and women in U.K. (3.97%). In 2019, ASMR in men and women was lowest in U.K. [men: 6.14 (5.57, 6.47) per 100,000 population; women: 4.69 (4.07, 5.07)]. Highest ASMR in men and women was both in U.S. [men: 21.45 (19.84, 22.43) per 100,000 population; women: 15.11 (13.41, 16.57) per 100,000 population], which were about three times those in U.K.

### The trends of CKD DALY

**Figure 5:**
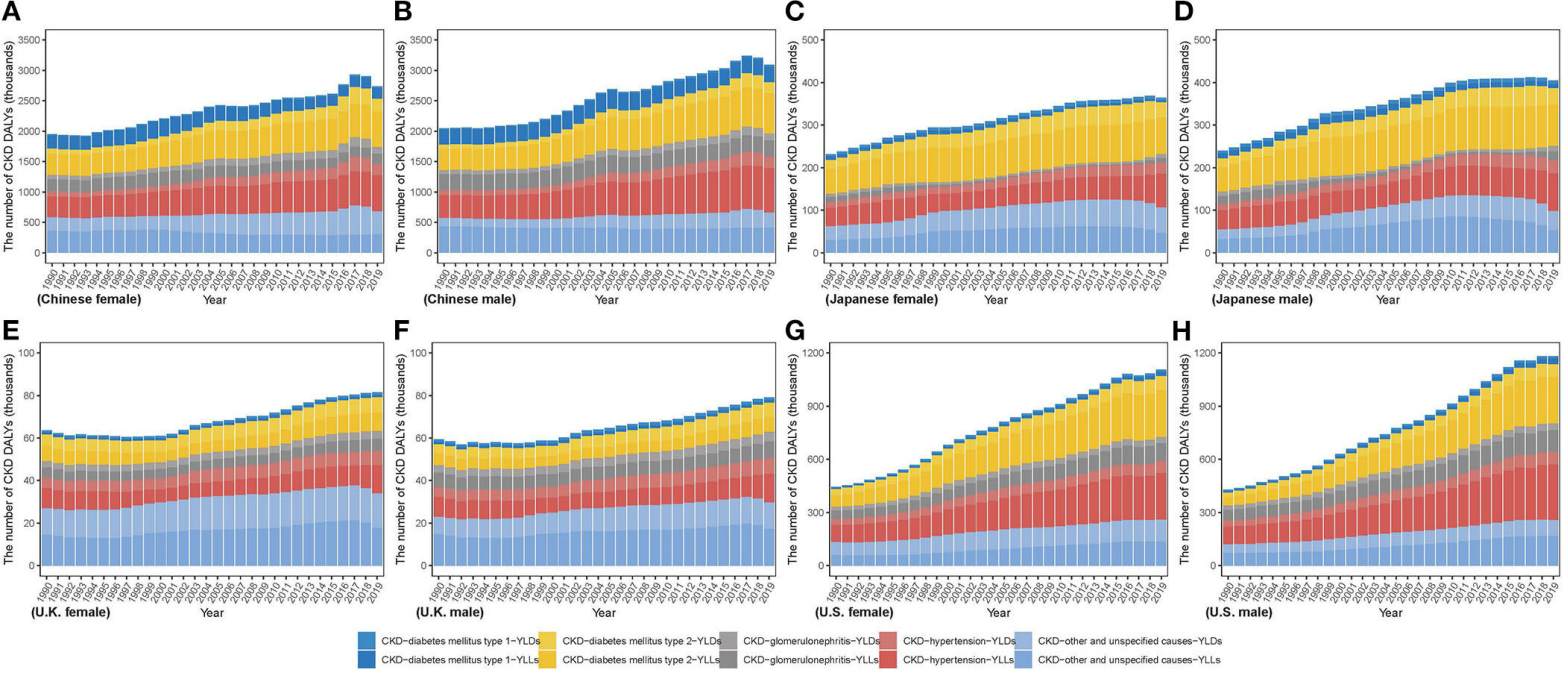
The number of CKD disability-adjusted life years from 1990 to 2019 in **(A)** Chinese women **(B)** Chinese men **(C)** Japanese women **(D)** Japanese men **(E)** U.K. women **(F)** U.K. men **(G)** U.S. women **(H)** U.S. men.

As Figure 5 describes, men in China and Japan had more DALY number, whereas women in U.K. had more between 1990 and 2019. In U.S., although women had more number in 1990, the number in men has gradually increased by more percent and surpassed that in women. From 1990 to 2019, DALY number in men and women increased in all countries by various percents, ranging from 33.19% and 27.98% in U.K. to 176.1% and 148.41% in U.S., respectively. In 2019, men accounted for more DALY number in four countries except for U.K. [men: 79,117.16 (69,918.32, 89,695.43); women: 81,447.62 (70,746.63, 93,171.63)]. Meanwhile, highest DALY number in men and women was both in China [men: 3,093,165.10 (2,554,295.46, 3,699,310.64); women: 2,738,678.28 (2,277,232.85, 3,240,170.77)]; while lowest both in U.K. [men: 79,117.1 (69,918.32, 89,695.43); women: 81,447.62 (70,746.63, 93,171.63)]. DALY number in men and women was over thirty times higher in China than in U.K. Notably, CKD due to diabetes and hypertension account a lower proportion for incidence to total CKD but a higher proportion for deaths and DALYs.

**Figure 6:**
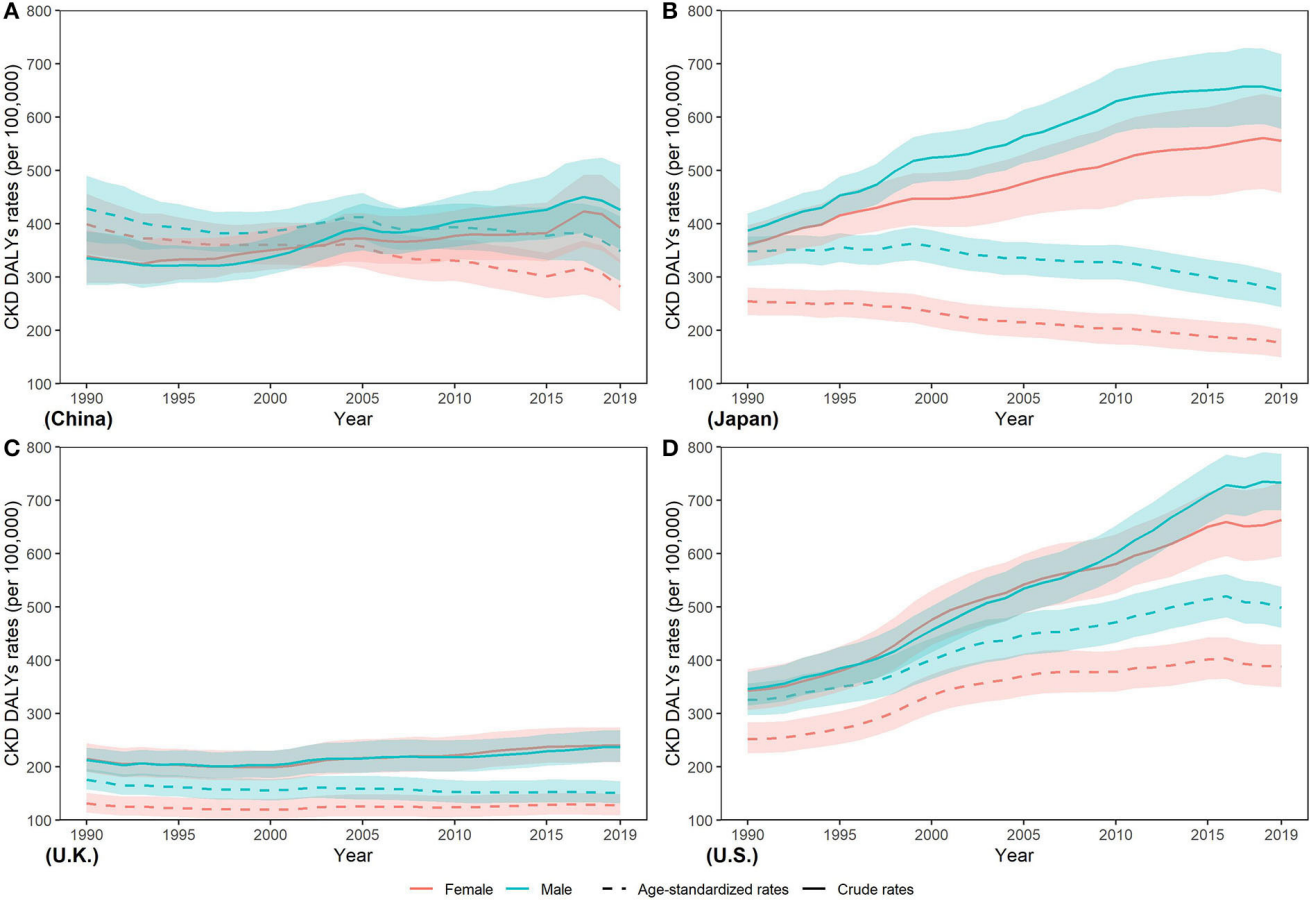
Trends in CKD disability-adjusted life year rates from 1990 to 2019 in **(A)** China **(B)** Japan **(C)** U.K., and **(D)** the U.S.

Between 1990 and 2019, a higher age standardized DALY rate (ASDR) in men was shown in those four countries (Figure 6). Additionally, from 1990 to 2019, ASDR decreased in four countries except for the increase in U.S. (men: 53.26%; women: 54.07%). In 2019, lowest ASDR in both sexes was observed in U.K. (men: 151.09 (132.16, 173.23) per 100,000 population; women: 127.85 (109.49, 149.29)] while the highest in U.S. [men: 498.84 (460.79, 537.79) per 100,000 population; women: 388.36 (350.06, 429.6) per 100,000 population]. Highest ASDR was about two times the lowest.

**Figure 7:**
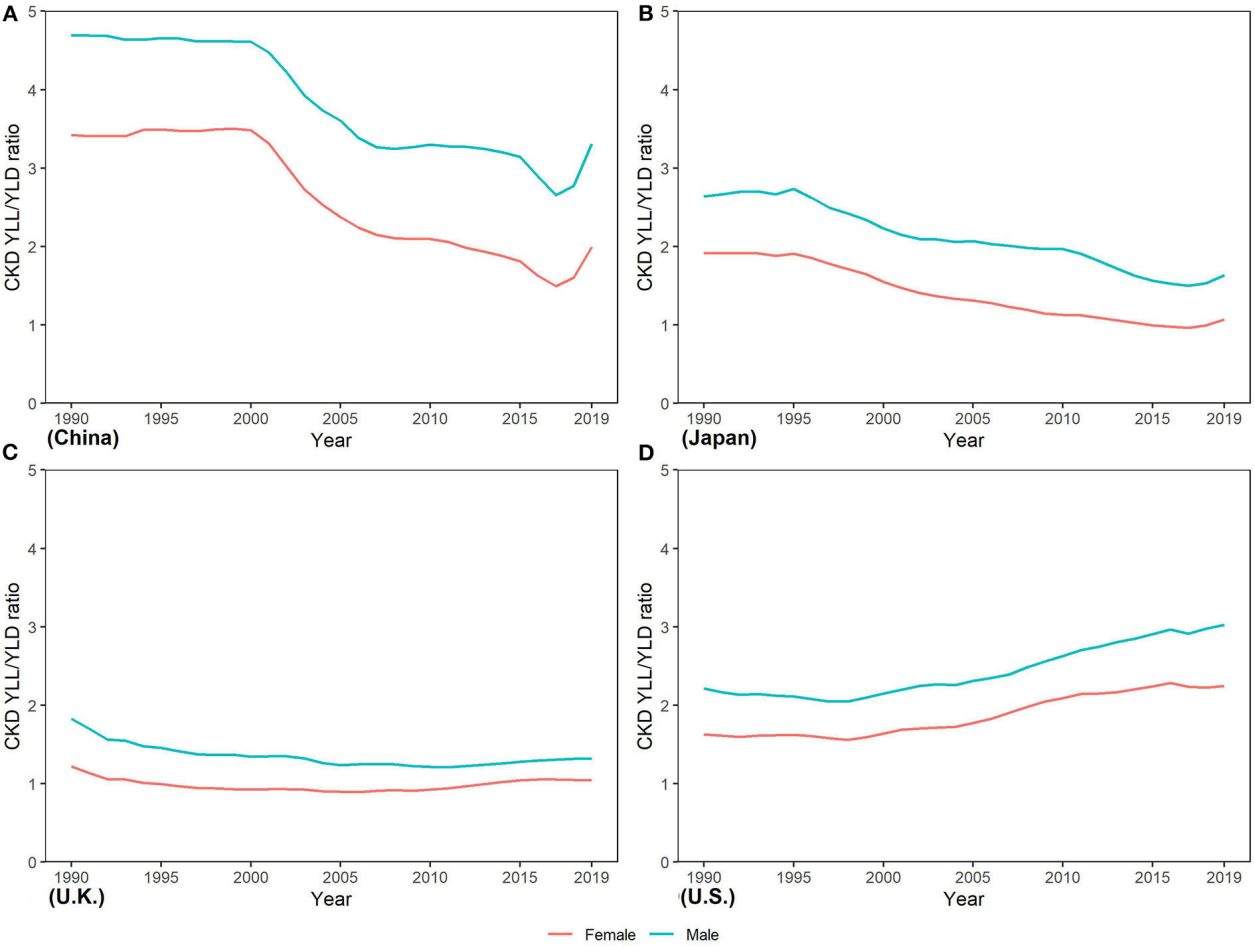
Trends in years of life lost/years of the life disability ratio of CKD from 1990 to 2019 in **(A)** China **(B)** Japan, **(C)** U.K., and **(D)** the U.S.

The YLL/YLD ratio is an important indicator for a GBD study. From Figure 7, we can see that the YLL/YLD ratio declined from 1990 to 2019 in the four countries except U.S. (2.20 to 3.29 in men and 1.77 to 2.59 in women), and China has the largest decline (4.85 to 3.19 in men and 3.33 to 2.09 in women), which means that the proportion of YLL in the burden of CKD in U.S. continues to rise over time. In 2019, the YLL/YLD ratio was far > 1 in China, Japan, and U.S., while it was close to 1 in U.K. In addition, we noticed that the YLL/YLD ratio was higher in men than in women in all four countries.

### Decomposition of the difference in CKD DALYs between 1990 and 2019

**Figure 8:**
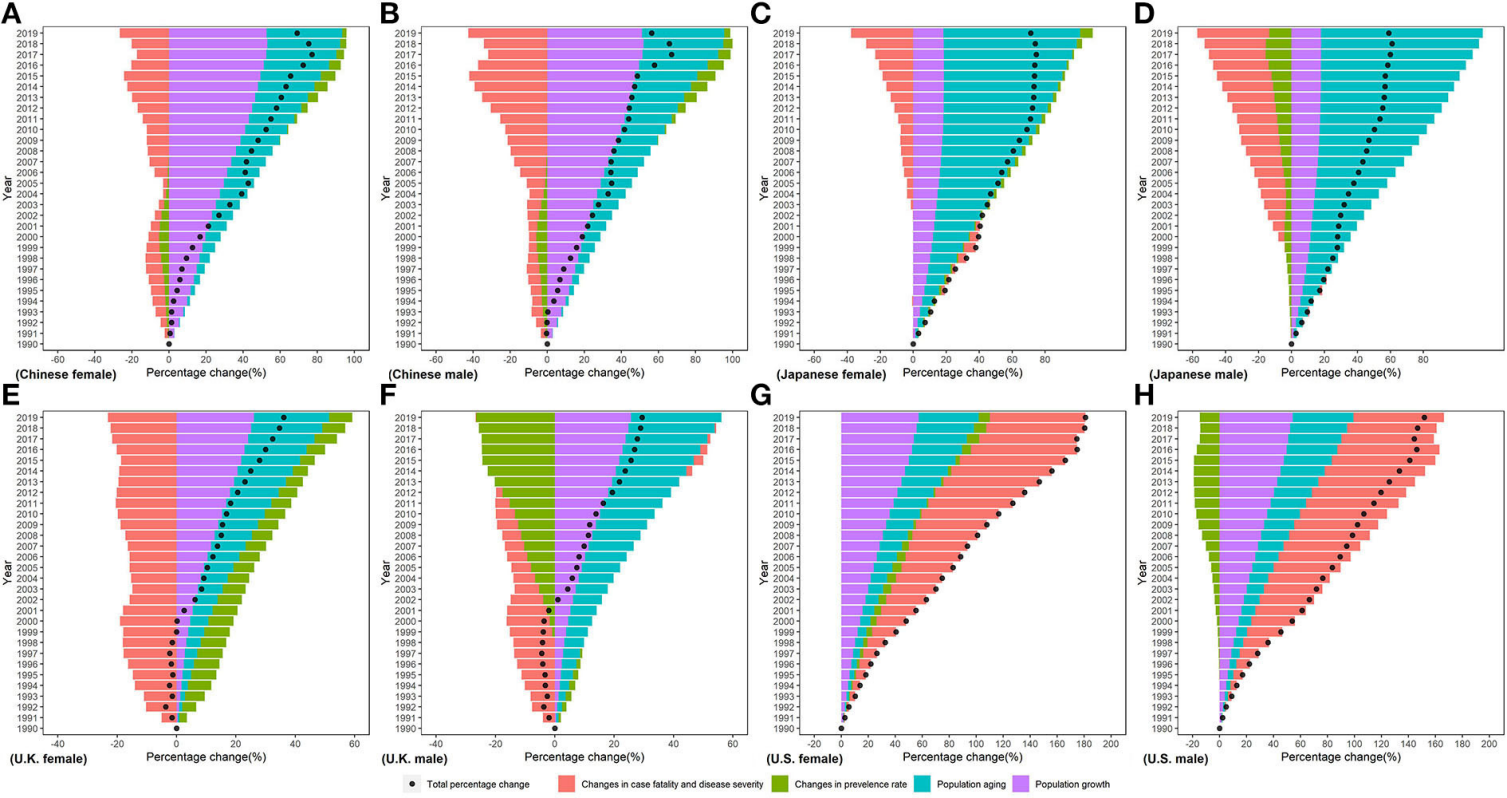
Percentage change in CKD disability-adjusted life years by sex in **(A)** Chinese female, **(B)** Chinese male, **(C)** Japanese female, **(D)** Japanese male, **(E)** U.K. female, **(F)** U.K. male, **(G)** U.S. female, and **(H)** U.S. male in 199096-2019 due to population growth, changes in population age structures, changes in the prevalence rate, changes in case fatality, and disease severity.

Difference in absolute number of DALYs from 1990 to 2019 was the result of changes in four underlying components: (1) population growth; (2) population aging; (3) prevalence rate; (4) case fatality and disease severity (see in Figure 8). Population growth accounted for increases in numbers of DALYs across all countries, but its contribution ranged from 17.82% in Japanese women to 52.68% in Chinese men. Population aging led to increasing DALYs in four countries, but its relative contribution ranged from 25.30% in U.K. men to 98.21% in Japanese women. Contribution of changes in the prevalence rate to DALYs of CKD in Chinese was negative before 2005 and positive after 2005. Changes in the prevalence rate drove increase in CKD DALYs in men and a decrease in CKD DALYs in women in Japan, U.K., and U.S. The relative contribution of changes in the prevalence rate ranged from 26.47% in U.K. women to 8.18% in U.S. men. Between 1990 and 2019, changes in case fatality and disease severity led to a decrease for DALY of CKD in Chinese, Japanese, and U.K. men; little contribution to the change in DALY of CKD in U.K. men; and an increase in DALY of CKD in U.S. The contribution of changes in case fatality and disease severity to changes in DALY of CKD ranged from −43.51% in Japanese women to 70.92% in U.S. men.

## Discussion

In this study, we performed a comprehensive estimation on the trend of CKD incidence, mortality, and DALY by sex from 1990 to 2019 in China, Japan, U.K., and U.S. From 1990 to 2019, CKD DALYs increased in all four countries, with population growth and population aging contributing significantly. More serious disease severity distribution and higher case fatality rates in US led an increase in CKD DALYs. Between 1990 and 2019, the ASMR and ASDR of CKD increased in US and decreased in China, Japan, and UK. The YLL/YLD ratio increased in US, indicating that the proportion of YLL in the total disease burden increased. And the YLL/YLD ratio was higher in men than in women in all four countries.

From 1990 to 2019, number of incidence cases, death, and DALY were all on the growth by various degrees. After age standardization of the rate, ASIR still showed a slightly increasing trend in four countries. Hypertension and diabetes are growing non-communicable diseases and important risk factors in CKD (22). In the past several years, the increasing incidence of hypertension and diabetes has led to an increasing incidence of CKD (4). There were also many risk factors associated with development of CKD (23). Many studies found that obesity (24), an unhealthy dietary pattern (25, 26), and unproper water drinking (27) could be associated with greater decrease in kidney function and even lead to CKD. Moreover, these risk factors were more common in public than that in the past (28). These variations may partly result to the increase in ASIR in four countries. Furthermore, there was disparity in the burden of CKD between men and women. A higher ASIR in women occurred in four countries except for Japan. A previous study has suggested that CKD was likely to affect more women than men except for Japan (29), which led to higher ASIR in women except for Japan. Moreover, pregnancy in women may give them additional opportunities to screen for CKD. In Japan, a reversal situation was observed that higher ASIR occurred in men. Many studies based on Japanese supported our results in this study (30, 31). A research has found that the association between hypertension and the risk of CKD was weaker in women than in men (30). Meanwhile, another study demonstrated that higher body mass index (BMI) in Japanese was associated with higher odds of CKD in men, not in women (31). Based on difference in association between hypertension, higher BMI and the risk of CKD, men in Japan had a relative higher ASIR compared with women.

Although there was an increasing trend in ASIR of CKD, ASMR was on the decreasing trend in four countries, except for U.S. In the prevention of CKD mortality, we have not seen so much progress as in many other important non-communicable diseases. From 1990 to 2019, the global age-standardized mortality rate declined by 32.34% in cardiovascular disease, 15.22% for cancer, 41.74% for COPD, but rose by 13.33% for CKD (2). However, numbers of death were increasing, which may be partly due to population growth and demographic aging. With rapid economic and medical improvement, better therapy such as renal replacement was used for the treatment in patients with CKD, especially in patients with end stage renal disease (ESRD). Survival of patients with ESRD has gradually increased after renal replacement in the past decades, and efforts to reduce the mortality of patients improved (32). However, ASMR of CKD was still high globally. The awareness of CKD was quite low in the public (33) awareness rate with fewer than 10% was observed among patients with CKD in both developing (34) and developed countries (35), which may lead to severe complications later in life. Studies demonstrated that earlier evaluation of patients with CKD could slow the progression of the disease (36), while the late recognition of CKD would increase renal failure (37). Despite of high ASMR, decline of ASMR accounted for the majority of decline in ASMR in China, Japan, and U.K. However, rather than decline in ASMR as same as that in other three countries, ASMR in U.S. actually showed an increasing trend and higher than other three countries in 2019. In the U.S., although great medical therapy existed.

Great medical treatments exist in U.S., however, the U.S. health care system was unaffordable, unsustainable, and inaccessible to many people (38, 39), which resulted deaths due to failure to get medical treatment in time. Meanwhile, many people died due to the more discarded kidney. More than 150,000 kidneys were donated in the U.S. between 2004 and 2014, 17.9% of which were discarded. Compared with that in the U.S., 9% of the donated kidneys were discarded in France during the same period (40). A less-than-perfect kidney transplant also performed better than dialysis for patients in urgent need of kidney replacement. Additionally, due to many discarded kidney and highest standardized rates of ESRD in the U.S. (13), the U.S. holds the highest ASMR of CKD in four countries. Furthermore, there were differences of ASMR between men and women in four countries that ASMR was higher in men, although there was higher ASIR of CKD in women, leading to higher YLL/YLD ratios in men. This finding was consistent with the results in the previous study. Ricardo AC found that, due to factors such as drinking, smoking et al., men seem to have faster progression of CKD compared with women (41).

Our research shows that the incidence of CKD due to diabetes and hypertension accounts for a low proportion of the total CKD, but a high proportion of deaths and DALYs. This is mainly because patients with CKD with uncontrolled diabetes or hypertension can easily and quickly develop into patients with ESRD (22). Diabetes is now the main cause of ESRD worldwide (42, 43). Over the past decade, the United States Renal Data System (URSDS) data have shown that the number of diabetic patients entering the ESRD has gradually increased, which is closely related to the global pandemic of type 2 diabetes. The number of adults with diabetes quadrupled between 1980 and 2019 (from 108 million in 1980 to 463 million in 2019) (7). The increase in burden of diabetes holds a more steeply trend in developing countries. According to URSDS data, 46.16% of ERDS incident patients had diabetes in 2019, while glomerulonephritis, cystic kidney disease, and hypertension have remained relatively stable as the causes of ESRD (44). In the long run, strengthening public health education, reducing excessive body weight, regular exercise, and dietary approaches should reduce the increase in patients with diabetes (45), and these patients will become the major future pool of ESRD cases.

Decomposition analyses showed that the increase in CKD DALYs from 1990 to 2019 was primarily driven by population growth and aging. In fact, the underlying epidemiologic trends of CKD, such as incidence and death rates, have lessened but far from the offset of the impact of population growth and aging. As the aging continues (The proportion of people over the age of 65 is estimated to reach 11.7% in 2030) (46), the prevention and control of CKD will face greater challenges in the future. Change in the prevalence rate between 1990 and 2019 had a negative contribution to the disease burden of CKD in U.K., which is consistent with the estimation of GBD2019 (18). In fact, U.K. is one of the top 10 countries with the lowest DALY rates for CKD worldwide (11). Early identification of patients with CKD in the U.K. healthcare system (47) may be one of the reasons why the disease burden of CKD is well controlled. Changes in case fatality rate and disease severity (representing the average disease burden of a single case) positively contribute to the increased disease burden of CKD in the U.S., and this contribution increases year by year. This is also shown in the increasing YLL/YLD ratio in the U.S.

The YLL/YLD ratio can reflect the relative magnitude of the burden of death and disability caused by CKD, and help public health professionals identify the key points of CKD prevention and control. The YLL/YLD ratio of CKD in U.K. is much lower than that of China, Japan, and U.S. This is mainly due to the low CKD death rate in U.K. Both the YLL/YLD ratios of China and Japan declined rapidly from 1990 to 2019, which is related to the development of medical technology and economic levels. However, the ratio of YLL/YLD in U.S. has been rising over time, which means that more patients with CKD would die rather than live with the disability. ESRD, which has a high mortality rate, is an important process of CKD death, and its treatment requires expensive costs (48). Approximately nine of ten of treated patients with ESRD come from more developed countries that can still afford the cost of renal replacement therapy. There is a close relationship between the economic level of the country and the availability of renal replacement therapy, and the less developed countries cannot meet the growing demand for renal replacement therapy (45). The different YLL/YLD ratios in countries highlight the importance of improving the management of CKD risk factors at the primary care level and the need to expand access to affordable renal replacement services for patients with ESRD (48). In the long run, it is imperative to shift the global treatment of CKD from the treatment of ESRD to more active primary and secondary prevention. Health education, early kidney disease detection for CKD, and the implementation of renal protection treatment can be implemented at a low cost, which will substantially reduce the burden of ESRD and CKD (9).

Although this study estimated the difference in burden of CKD between four countries from 1990-2019, it still had several limitations. Due to analyzed data from GBD 2019, all limitations of the GBD 2019 methods outlined elsewhere also apply to our study (17, 18). Additionally, despite many methods and processes used to reduce the bias in the GBD study, it remained difficult to avoid the inaccuracy.

## Conclusion

From 1990 to 2019, CKD incidences, deaths, and DALYs all increased in China, Japan, U.K., and the U.S. The increase in the burden of CKD not only comes from population growth and aging but is also closely related to the prevalence of diabetes and hypertension. The average disease burden per case in the U.S. increased between 1990 and 2019, with an increasing proportion of death-related disease burden. Considering the high treatment costs of CKD and ESRD, it is urgent and necessary to transform CKD treatment into primary and secondary prevention.

## Data availability statement

The original contributions presented in the study are included in the article/Supplementary material, further inquiries can be directed to the corresponding author.

## Author contributions

CY and HW supervised the study. CY, HW, and DY designed the study. HW and CX collected and organized the data, analyzed the data, and interpreted the results. DY and HW wrote the first draft. CY, HW, DY, CX, FS, YL, and JZ reviewed and edited the manuscript. All authors contributed to the final draft and have approved the submitted version.

## Funding

This work was funded by National Natural Science Foundation of China (Grant Nos. 82173626 and 81773552) and Health Commission of Hubei Province Scientific Research Project (Grant No. WJ2019H304). The funders had no role in the study design, data collection, analysis, decision to publish, or preparation of the manuscript.

## Conflict of interest

The authors declare that the research was conducted in the absence of any commercial or financial relationships that could be construed as a potential conflict of interest.

## Publisher's note

All claims expressed in this article are solely those of the authors and do not necessarily represent those of their affiliated organizations, or those of the publisher, the editors and the reviewers. Any product that may be evaluated in this article, or claim that may be made by its manufacturer, is not guaranteed or endorsed by the publisher.
