# Supplementary material for: Comparison of trend in chronic kidney disease burden between China, Japan, the United Kingdom, and the United States
Source: Front Public Health. 2022 Sep 6;10:999848. doi: 10.3389/fpubh.2022.999848 (PMC9499831; doi:10.3389/fpubh.2022.999848)
Supplement: Supplementary file 1 [file Data_Sheet_1.docx]

Comparison of trend in chronic kidney disease burden between China, Japan, the United Kingdom, and the United States

**Supplementary Table 1(a)** The age-standardized rate of incidence, death, and DALY for CKD in China, Japan, U.K., and U.S. female.

|  |  | Incidence | | |  | Death | | |  | DALY | | |
| --- | --- | --- | --- | --- | --- | --- | --- | --- | --- | --- | --- | --- |
|  |  | 1990 | 2019 | Percent change (%) |  | 1990 | 2019 | Percent change (%) |  | 1990 | 2019 | Percent change (%) |
| **China** | |  |  |  |  |  |  |  |  |  |  |  |
|  | CKD | 152.72 (139.00,169.20) | 168.28 (154.63,183.25) | 10.19 |  | 12.07 (10.23,14) | 9.59  (7.76,11.45) | -20.55 |  | 399.81 (343.53,456.29) | 282.49 (235.82,332.93) | -29.34 |
|  | CKD due to diabetes mellitus type 1 | 0.61  (0.45,0.81) | 0.59  (0.45,0.78) | -3.28 |  | 1.03 (0.70,1.46) | 0.54  (0.36,0.79) | -47.57 |  | 43.68 (30.25,60.43) | 21.59 (14.73,29.81) | -50.57 |
|  | CKD due to diabetes mellitus type 2 | 17.96 (15.96,20.15) | 19.78 (17.91,21.91) | 10.13 |  | 3.71 (2.94,4.53) | 3.17  (2.43,3.95) | -14.56 |  | 96.43 (76.25,117.21) | 76.62 (60.35,93.78) | -20.54 |
|  | CKD due to glomerulonephritis | 3.68  (3.20,4.17) | 3.34  (2.92,3.81) | -9.24 |  | 1.11 (0.80,1.48) | 0.75  (0.52,1.07) | -32.43 |  | 49.87 (38.74,62.10) | 31.78 (24.35,40.17) | -36.27 |
|  | CKD due to hypertension | 10.89 (9.79,12.16) | 12.63 (11.48,13.89) | 15.98 |  | 3.96 (3.18,4.84) | 3.49  (2.69,4.36) | -11.87 |  | 95.93 (78.19,117.10) | 77.34 (61.98,94.61) | -19.38 |
|  | CKD due to other and unspecified causes | 119.59 (108.78,132.40) | 131.94 (121.08,143.62) | 10.33 |  | 2.25 (1.69,2.92) | 1.63  (1.14,2.22) | -27.56 |  | 113.89 (93.36,135.40) | 75.16 (59.48,92.05) | -34.01 |
| **Japan** | |  |  |  |  |  |  |  |  |  |  |  |
|  | CKD | 260.12 (237.27,283.91) | 260.17 (238.05,284.35) | 0.02 |  | 10.89 (9.61,11.65) | 6.87  (5.18,7.87) | -36.91 |  | 254.81 (229.49,280.94) | 176.78 (150.1,202.75) | -30.62 |
|  | CKD due to diabetes mellitus type 1 | 1.07  (0.85,1.34) | 1.21  (0.93,1.52) | 13.08 |  | 0.37 (0.24,0.55) | 0.17  (0.11,0.26) | -54.05 |  | 16.78 (11.16,23.11) | 11.09 (7.34,15.79) | -33.91 |
|  | CKD due to diabetes mellitus type 2 | 31.45 (28.35,34.68) | 30.81 (27.76,34.05) | -2.03 |  | 4.15 (3.33,4.97) | 2.56  (1.85,3.23) | -38.31 |  | 82.81 (68.76,96.22) | 55.03 (44.34,65.87) | -33.55 |
|  | CKD due to glomerulonephritis | 3.50  (3.10,3.95) | 3.41  (3.00,3.89) | -2.57 |  | 0.62 (0.45,0.85) | 0.34  (0.23,0.49) | -45.16 |  | 24.14 (19.63,29.65) | 16.81 (13.36,20.84) | -30.36 |
|  | CKD due to hypertension | 20.47 (18.65,22.42) | 20.82 (19.02,22.77) | 1.71 |  | 3.55 (2.73,4.32) | 2.39  (1.66,3.04) | -32.68 |  | 59.48 (48.26,70.98) | 41.17 (31.93,49.95) | -30.78 |
|  | CKD due to other and unspecified causes | 203.62 (186.22,221.75) | 203.92 (186.47,222.64) | 0.15 |  | 2.21 (1.49,3.04) | 1.41  (0.90,1.97) | -36.20 |  | 71.59 (56.62,87.43) | 52.68 (41.77,64.90) | -26.41 |
| **U.K.** | |  |  |  |  |  |  |  |  |  |  |  |
|  | CKD | 215.40 (194.58,238.46) | 222.31 (199.66,245.81) | 3.21 |  | 4.51 (4.13,4.74) | 4.69  (4.07,5.07) | 3.99 |  | 131.68 (114.83,151.11) | 127.85 (109.49,149.29) | -2.91 |
|  | CKD due to diabetes mellitus type 1 | 0.80  (0.68,0.93) | 0.85  (0.72,1.00) | 6.25 |  | 0.12 (0.07,0.19) | 0.10  (0.06,0.16) | -16.67 |  | 5.37  (3.74,7.42) | 4.87  (3.35,6.60) | -9.31 |
|  | CKD due to diabetes mellitus type 2 | 25.99 (23.18,29.07) | 26.59 (23.55,29.63) | 2.31 |  | 0.88 (0.65,1.20) | 0.88  (0.63,1.19) | 0.00 |  | 23.07 (17.91,29.14) | 22.34 (17.35,28.48) | -3.16 |
|  | CKD due to glomerulonephritis | 2.56  (2.28,2.88) | 2.82  (2.50,3.17) | 10.16 |  | 0.52 (0.37,0.70) | 0.51  (0.35,0.71) | -1.92 |  | 19.55 (16.11,23.63) | 18.68 (15.19,22.64) | -4.45 |
|  | CKD due to hypertension | 17.59 (15.88,19.41) | 18.05 (16.21,19.95) | 2.62 |  | 1.30  (1.00,1.62) | 1.47  (1.09,1.83) | 13.08 |  | 25.90 (21.09,30.96) | 26.76  (21.84,32.00) | 3.32 |
|  | CKD due to other and unspecified causes | 168.45 (152.37,186.12) | 174.00 (156.38,192.18) | 3.29 |  | 1.69 (1.35,2.02) | 1.73  (1.35,2.11) | 2.37 |  | 57.77  (49.00,67.30) | 55.20 (45.87,64.95) | -4.45 |
| **U.S.** | |  |  |  |  |  |  |  |  |  |  |  |
|  | CKD | 316.39 (289.61,346.06) | 335.44 (309.59,364.23) | 6.02 |  | 8.45 (7.68,8.94) | 15.11 (13.41,16.57) | 78.82 |  | 252.07 (225.87,283.64) | 388.36 (350.06,429.6) | 54.07 |
|  | CKD due to diabetes mellitus type 1 | 1.56  (1.30,1.87) | 1.49  (1.26,1.76) | -4.49 |  | 0.18 (0.11,0.27) | 0.39  (0.25,0.59) | 116.67 |  | 9.09  (6.23,12.61) | 15.68 (10.51,21.68) | 72.50 |
|  | CKD due to diabetes mellitus type 2 | 37.56 (33.84,41.63) | 40.52 (36.78,44.64) | 7.88 |  | 1.93 (1.43,2.50) | 5.00  (3.89,6.22) | 159.07 |  | 55.42 (43.52,67.44) | 113.42 (92.05,135.88) | 104.66 |
|  | CKD due to glomerulonephritis | 4.78  (4.23,5.36) | 4.56  (4.09,5.09) | -4.60 |  | 1.26 (0.93,1.67) | 1.63  (1.20,2.20) | 29.37 |  | 43.84 (36.76,52.81) | 59.10 (47.92,72.44) | 34.81 |
|  | CKD due to hypertension | 24.23 (22.14,26.48) | 25.97 (23.94,28.25) | 7.18 |  | 3.39 (2.75,3.99) | 5.59  (4.44,6.71) | 64.90 |  | 66.92 (56.56,77.19) | 103.43 (86.09,121.29) | 54.56 |
|  | CKD due to other and unspecified causes | 248.26 (227.51,271.34) | 262.90 (242.89,284.91) | 5.90 |  | 1.70 (1.21,2.29) | 2.49  (1.76,3.42) | 46.47 |  | 76.80 (61.89,92.11) | 96.72 (76.75,118.65) | 25.94 |

Abbreviation: CKD, chronic kidney disease; DALY, disability-adjusted life years; UI: uncertainly interval.

**Supplementary Table 1(b)** The age-standardized rate of incidence, death, and DALY for CKD in China, Japan, U.K., and U.S. male.

|  |  | Incidence | | |  | Death | | |  | DALY | | |
| --- | --- | --- | --- | --- | --- | --- | --- | --- | --- | --- | --- | --- |
|  |  | 1990 | 2019 | Percent change (%) |  | 1990 | 2019 | Percent change (%) |  | 1990 | 2019 | Percent change (%) |
| **China** | |  |  |  |  |  |  |  |  |  |  |  |
|  | CKD | 141.33 (128.09,156.06) | 156.51  (142.15,172.39) | 10.74 |  | 14.82  (12.59,17.37) | 13.92  (11.61,16.55) | -6.07 |  | 428.58  (366.93,490.16) | 348.96  (292.80,413.96) | -18.58 |
|  | CKD due to diabetes mellitus type 1 | 0.95  (0.74,1.21) | 1.01  (0.80,1.27) | 6.32 |  | 1.07  (0.72,1.43) | 0.74  (0.49,1.09) | -30.84 |  | 46.06  (31.51,62.41) | 30.25  (20.71,43.12) | -34.32 |
|  | CKD due to diabetes mellitus type 2 | 21.38  (19.01,23.97) | 23.21  (20.78,25.91) | 8.56 |  | 4.37  (3.44,5.45) | 4.27  (3.29,5.41) | -2.29 |  | 100.95  (79.20,124.17) | 91.64  (71.98,113.97) | -9.22 |
|  | CKD due to glomerulonephritis | 6.94  (6.27,7.73) | 6.79  (6.09,7.64) | -2.16 |  | 1.53  (1.14,2.01) | 1.28  (0.89,1.74) | -16.34 |  | 59.57  (46.25,75.48) | 43.85  (34.20,55.24) | -26.39 |
|  | CKD due to hypertension | 13.58  (12.19,15.11) | 15.76  (14.23,17.40) | 16.05 |  | 5.01  (4.00,6.21) | 5.15  (4.13,6.33) | 2.79 |  | 110.31  (89.40,133.53) | 103.47  (83.82,127.55) | -6.20 |
|  | CKD due to other and unspecified causes | 98.48  (89.13,108.85) | 109.75  (99.76,120.84) | 11.44 |  | 2.85  (2.08,3.80) | 2.49  (1.70,3.48) | -12.63 |  | 111.69  (91.01,134.29) | 79.75  (62.61,101.12) | -28.60 |
| **Japan** | |  |  |  |  |  |  |  |  |  |  |  |
|  | CKD | 287.64  (266.43,311.62) | 322.81  (296.77,351.25) | 12.23 |  | 16.00  (14.92,16.57) | 11.59  (10.04,12.55) | -27.56 |  | 348.68  (321.69,376.56) | 275.45  (243.81,306.95) | -21.00 |
|  | CKD due to diabetes mellitus type 1 | 2.10  (1.68,2.58) | 2.45  (1.97,3.06) | 16.67 |  | 0.56  (0.37,0.83) | 0.36  (0.23,0.54) | -35.71 |  | 24.03  (15.77,34.17) | 18.05  (11.56,25.79) | -24.89 |
|  | CKD due to diabetes mellitus type 2 | 45.11  (41.06,49.88) | 50.26  (45.43,55.63) | 11.42 |  | 5.64  (4.59,6.75) | 4.04  (3.20,4.93) | -28.37 |  | 110.94  (92.28,129.39) | 86.91  (71.18,103.13) | -21.66 |
|  | CKD due to glomerulonephritis | 10.07  (9.08,11.19) | 10.43  (9.37,11.62) | 3.57 |  | 1.21  (0.86,1.64) | 0.82  (0.58,1.13) | -32.23 |  | 40.43  (32.64,49.77) | 31.06  (24.58,38.36) | -23.18 |
|  | CKD due to hypertension | 28.53  (26.22,31.08) | 32.64  (29.90,35.47) | 14.41 |  | 5.30  (4.22,6.35) | 3.98  (3.05,4.84) | -24.91 |  | 90.10  (74.81,105.94) | 72.33  (59.61,84.92) | -19.72 |
|  | CKD due to other and unspecified causes | 201.83  (186.95,218.71) | 227.03  (208.88,246.44) | 12.49 |  | 3.30  (2.32,4.37) | 2.40  (1.65,3.19) | -27.27 |  | 83.17  (67.07,101.56) | 67.09  (53.46,81.45) | -19.33 |
| **U.K.** | |  |  |  |  |  |  |  |  |  |  |  |
|  | CKD | 185.40  (167.04,205.18) | 208.92  (189.22,231.62) | 12.69 |  | 7.23  (6.80,7.46) | 6.14  (5.57,6.47) | -15.08 |  | 176.01  (158.08,196.28) | 151.09  (132.16,173.23) | -14.16 |
|  | CKD due to diabetes mellitus type 1 | 1.43  (1.23,1.67) | 1.53  (1.30,1.77) | 6.99 |  | 0.16  (0.10,0.26) | 0.12  (0.07,0.18) | -25.00 |  | 6.95  (4.81,9.61) | 5.64  (3.88,7.74) | -18.85 |
|  | CKD due to diabetes mellitus type 2 | 28.40  (25.16,31.77) | 31.98  (28.53,35.81) | 12.61 |  | 1.14  (0.82,1.56) | 0.94  (0.67,1.31) | -17.54 |  | 26.84  (20.52,33.83) | 23.70  (18.15,30.43) | -11.70 |
|  | CKD due to glomerulonephritis | 5.75  (5.13,6.43) | 6.26  (5.57,6.98) | 8.87 |  | 1.04  (0.73,1.42) | 0.84  (0.58,1.17) | -19.23 |  | 31.37  (26.03,37.75) | 26.29  (21.48,31.61) | -16.19 |
|  | CKD due to hypertension | 19.71  (17.69,21.90) | 22.35  (20.16,24.68) | 13.39 |  | 2.32  (1.85,2.83) | 2.09  (1.63,2.58) | -9.91 |  | 41.14  (34.57,48.56) | 36.98  (30.65,43.92) | -10.11 |
|  | CKD due to other and unspecified causes | 130.09  (117.34,144.04) | 146.8  (132.86,162.58) | 12.84 |  | 2.57  (2.00,3.13) | 2.14  (1.63,2.62) | -16.73 |  | 69.71  (59.29,80.16) | 58.49  (49.12,68.17) | -16.10 |
| **U.S.** | |  |  |  |  |  |  |  |  |  |  |  |
|  | CKD | 283.88  (257.36,314.43) | 300.27  (275.12,327.33) | 5.77 |  | 12.55  (11.74,12.98) | 21.45  (19.84,22.43) | 70.92 |  | 325.48  (296.91,356.66) | 498.84  (460.79,537.79) | 53.26 |
|  | CKD due to diabetes mellitus type 1 | 2.95  (2.49,3.55) | 2.93  (2.52,3.45) | -0.68 |  | 0.22  (0.14,0.33) | 0.54  (0.33,0.81) | 145.45 |  | 11.88  (7.99,16.83) | 21.47  (14.03,30.08) | 80.72 |
|  | CKD due to diabetes mellitus type 2 | 43.15  (38.44,48.57) | 45.99  (41.43,50.94) | 6.58 |  | 1.81  (1.30,2.45) | 6.02  (4.61,7.59) | 232.60 |  | 54.87  (41.85,69.15) | 133.22  (104.83,160.42) | 142.79 |
|  | CKD due to glomerulonephritis | 10.81  (9.55,12.20) | 10.73  (9.56,11.97) | -0.74 |  | 2.08  (1.52,2.74) | 2.29  (1.69,3.09) | 10.10 |  | 64.14  (53.06,77.03) | 73.76  (59.85,91.97) | 15.00 |
|  | CKD due to hypertension | 28.40  (25.64,31.35) | 30.32  (27.60,33.07) | 6.76 |  | 5.40  (4.46,6.28) | 8.70  (7.07,10.3) | 61.11 |  | 102.33  (87.74,117.09) | 156.47  (131.88,184.12) | 52.91 |
|  | CKD due to other and unspecified causes | 198.57  (180.03,219.83) | 210.30  (192.46,229.13) | 5.91 |  | 3.04  (2.18,4.06) | 3.90  (2.75,5.16) | 28.29 |  | 92.25  (75.06,111.71) | 113.92  (90.75,140.23) | 23.49 |

Abbreviation: CKD, chronic kidney disease; DALY, disability-adjusted life years; UI: uncertainly interval.

**GATHER checklist: Checklist of information that should be included in new reports of global health estimates**

| Item # | Checklist item | Reported on page # |
| --- | --- | --- |
| Objectives and funding | | |
| 1 | Define the indicator(s), populations (including age, sex, and geographic entities), and time period(s) for which estimates were made. | #3 Main text (Methods) |
| 2 | List the funding sources for the work. | #9 Main text (Funding) |
| Data Inputs | | |
| *For all data inputs from multiple sources that are synthesized as part of the study:* | | |
| 3 | Describe how the data were identified and how the data were accessed. | #3 Main text (Methods) |
| 4 | Specify the inclusion and exclusion criteria. Identify all ad-hoc exclusions. | #3 Main text (Methods) |
| 5 | Provide information on all included data sources and their main characteristics. For each data source used, report reference information or contact name/institution, population represented, data collection method, year(s) of data collection, sex and age range, diagnostic criteria or measurement method, and sample size, as relevant. | Online data citation tools: https://vizhub.healthdata.org/gbd-results/ |
| 6 | Identify and describe any categories of input data that have potentially important biases (e.g., based on characteristics listed in item 5). | #3 Main text (Methods) |
| *For data inputs that contribute to the analysis but were not synthesized as part of the study:* | | |
| 7 | Describe and give sources for any other data inputs. | https://vizhub.healthdata.org/gbd-results/ |
| *For all data inputs:* | | |
| 8 | Provide all data inputs in a file format from which data can be efficiently extracted (e.g., a spreadsheet rather than a PDF), including all relevant meta-data listed in item 5. For any data inputs that cannot be shared because of ethical or legal reasons, such as third-party ownership, provide a contact name or the name of the institution that retains the right to the data. | https://vizhub.healthdata.org/gbd-results/ |
| Data analysis | | |
| 9 | Provide a conceptual overview of the data analysis method. A diagram may be helpful. | #3,4 Main text (Methods) |
| 10 | Provide a detailed description of all steps of the analysis, including mathematical formulae. This description should cover, as relevant, data cleaning, data pre-processing, data adjustments and weighting of data sources, and mathematical or statistical model(s). | #3,4 Main text (Methods) |
| 11 | Describe how candidate models were evaluated and how the final model(s) were selected. | #3,4 Main text (Methods) |
| 12 | Provide the results of an evaluation of model performance, if done, as well as the results of any relevant sensitivity analysis. | #3,4 Main text (Methods) |
| 13 | Describe methods for calculating uncertainty of the estimates. State which sources of uncertainty were, and were not, accounted for in the uncertainty analysis. | #3,4 Main text (Methods) |
| 14 | State how analytic or statistical source code used to generate estimates can be accessed. | http://ghdx.healthdata.org/gbd-2019-code |
| Results and Discussion | | |
| 15 | Provide published estimates in a file format from which data can be efficiently extracted. | Main text, appendix |
| 16 | Report a quantitative measure of the uncertainty of the estimates (e.g. uncertainty intervals). | Main text, appendix |
| 17 | Interpret results in light of existing evidence. If updating a previous set of estimates, describe the reasons for changes in estimates. | Main text (Methods and Discussion) |
| 18 | Discuss limitations of the estimates. Include a discussion of any modelling assumptions or data limitations that affect interpretation of the estimates. | Main text (Limitations) |
